# Feline Milk-Derived *Lactiplantibacillus plantarum* MNN and Galacto-Oligosaccharides Ameliorate Stress-Related Behavioral Abnormalities and Alter Tryptophan Metabolism in CSDS Mice

**DOI:** 10.3390/microorganisms14102185

**Published:** 2026-09-30

**Authors:** Xue Wang, Xinyu Gong, Shaohui Lang, Lu Li, PanPan Wang, Jinfa Chen, Jun Han, Zhengping Wang, Min Wen

**Affiliations:** 1 Shandong Key Laboratory of Applied Technology for Protein and Peptide Drugs, Institute of Biopharmaceutical Research, Liaocheng University, Liaocheng 252000, China; 15550778598@163.com (X.W.); pinkrose1998@163.com (X.G.);; 2 Pet Nutrition Research and Development Center, Gambol Pet Group Co., Ltd., Liaocheng 252000, China

**Keywords:** stress, *Lactiplantibacillus plantarum*, galacto-oligosaccharides, synbiotic, tryptophan metabolism

## Abstract

Chronic stress is a critical predisposing factor for neuropsychiatric and metabolic disorders, profoundly affecting both physical health and behavioral performance. Stress induces gut microbiota dysbiosis and disrupts tryptophan metabolism, notably via the kynurenine (KYN) and 5-hydroxytryptamine (5-HT) pathways. Targeted modulation of the gut–brain axis through probiotics and prebiotics has therefore emerged as a promising approach for preventing and alleviating stress-related disorders, yet the efficacy of specific synbiotic combinations remains largely unexplored. This study investigated the effects of feline milk-derived *Lactiplantibacillus plantarum* MNN, galacto-oligosaccharides (GOS), and their combination on stress-related behavioral abnormalities and tryptophan metabolism in a mouse model of chronic social defeat stress (CSDS). Behavioral outcomes, gut microbial composition, cecal tryptophan metabolites, and molecular markers associated with serotonin (5-HT) synthesis and neuroplasticity were evaluated. Intervention with MNN, GOS, and MNN + GOS attenuated several CSDS-induced behavioral abnormalities and was associated with alterations in gut microbial composition and cecal tryptophan metabolism. In the SIT, the social interaction ratio increased from 0.307 ± 0.089 in CSDS mice to 0.778 ± 0.042 (*p* = 0.003), 0.826 ± 0.035 (*p* = 0.001), 1.445 ± 0.114 (*p* < 0.0001) in the MNN, GOS, and MNN + GOS groups, respectively. Cecal indoxyl sulfate levels decreased from 1.944 ± 0.286 in CSDS mice to 0.206 ± 0.022 (*p* < 0.0001), 0.231 ± 0.058 (*p* < 0.0001) and 0.275 ± 0.018 (*p* < 0.0001) in the MNN, GOS, and MNN + GOS groups, respectively. The interventions also increased intestinal and hippocampal 5-HT signals and partially restored the expression of tryptophan hydroxylases, brain-derived neurotrophic factor (BDNF), and postsynaptic density protein 95 (PSD95). These findings suggest that MNN, GOS, and MNN + GOS interventions may alleviate stress-related abnormalities, potentially through modulation of the microbiota-tryptophan-5-HT axis. However, the causal role of the gut microbiota and the efficacy of these interventions in cats require further investigation.

## 1. Introduction

In recent years, cats have gained growing popularity as companion animals worldwide, with the global cat population reported to have reached 373 million by 2025 [1]. With growing attention to companion animal welfare, increasing emphasis has been placed on the emotional well-being of cats in addition to their physical health. Felines are naturally highly vigilant and fearful of unfamiliar stimuli [2]. However, in highly urbanized feline husbandry settings, a range of acute and chronic stressors including abrupt environmental changes, multi-cat households, and frequent social stimuli have emerged as key drivers of sustained pathological stress in domestic cats.

Chronic social defeat stress (CSDS) is a well-established model of chronic psychosocial stress that induces depression-like and anxiety-like behaviours through repeated social defeat [3]. Because chronic environmental and social stressors are also common triggers of stress-related disorders in cats, the CSDS model provides a suitable platform for investigating stress-associated physiological and behavioral changes in feline models, facilitating the translation of preclinical findings to clinical applications. Tryptophan serves as an essential biochemical precursor for the biosynthesis of 5-HT, and the central serotonergic system plays a critical role in regulating fear and anxiety-related states as well as stress responses [4]. Under chronic stress conditions, tryptophan metabolism is diverted toward the KYN pathway rather than the 5-HT synthetic pathway, resulting in impaired serotonergic signaling and contributing to stress-related behavioral abnormalities [5,6]. Accumulating evidence indicates that restoration of tryptophan–5-HT metabolic homeostasis effectively ameliorates CSDS-induced behavioral abnormalities [4]. Therefore, targeting tryptophan metabolic homeostasis may represent a promising strategy for alleviating stress-related disorders in cats.

Common clinical anti-stress agents like gabapentin have limited efficacy and carry side effects such as drowsiness. Because nutritional management has superior safety and tolerability compared with medications, developing anti-stress bioactive ingredients for cats an urgent priority [7]. Recent studies show that dietary nutrients including polyphenols, functional amino acids and probiotics can effectively reduce stress-related behaviors in cats [8]. A growing body of evidence has recently corroborated the antidepressant effects of probiotics [9,10]. *L. plantarum*, a widely studied probiotic species, has demonstrated potential health-promoting effects in cats by modulating gut microbiota composition, enhancing immune function, and improving antioxidant capacity [11]. Furthermore, previous studies have demonstrated that Lactobacillus supplementation alleviates stress responses and ameliorates depression-like behaviors in animal models [12], with *Lactobacillus plantarum* reported to exert beneficial effects on stress-related behavioral disorders, potentially via the modulation of serotonin-associated pathways [13]. Notably, probiotics derived from their original host are considered promising candidates due to their enhanced host adaptability, colonization potential, and functional compatibility. Our research group previously isolated a strain of *L. plantarum* from cat’s milk, which has been confirmed to possess good probiotic proper-ties and safety [14]. Nevertheless, the potential effects of MNN on emotional and stress-related behavioral abnormalities remain unclear. Synbiotics combine probiotics with prebiotics to enhance probiotic viability and functional efficacy [15]. As a classic prebiotic, the molecular structure of GOS features terminal lactose connected with repeating galactose units. GOS has been shown to promote intestinal health in cats by modulating faecal characteristics and supporting beneficial microbial populations [16]. In addition, GOS alleviates anxiety-like behaviors in mice [17]. Studies have demonstrated that synbiotic formulations combining *Lactobacillus plantarum* and GOS have synergistic effects in improving metabolic homeostasis [18], and ameliorating gastrointestinal disorders [19].

This study used a CSDS model to systematically evaluate the effects of the cat’s milk-derived probiotic *L. plantarum* MNN, and synbiotic (MNN + GOS) on stress-related behavioral abnormalities. The study focused on investigating whether these agents can alleviate CSDS-induced behavioral abnormalities in mice and elucidating the underlying mechanism with a particular focus on tryptophan metabolism.

## 2. Materials and Methods

### 2.1. Animals

Five-week-old male C57BL/6 mice (Shandong Pengyue Laboratory Animal Technology Co., Ltd., Jinan, Shandong, China) were purchased. Upon arrival, the mice were allocated into experimental groups with six mice per group, and all mice were singly housed throughout the entire experimental period. Retired male CD-1 breeding mice (4 months old) were purchased from Nanjing Huimiao Xin Biotechnology Co., Ltd. (Nanjing, China) and housed individually upon arrival. All mice were acclimatized in the animal housing facility (temperature 22–26 °C, humidity approximately 50%) for at least one week and maintained under a 12 h light–dark cycle (07:00–19:00), standard chow and drinking water were available ad libitum. This animal experiment was approved by the Ethics Committee for Laboratory Animals of the Institute of Biopharmaceutical Research, Liaocheng University (Liaocheng, Shandong, China; Approval No.: AP2024022959). The study was conducted in strict compliance with local laws, regulations and relevant institutional guidelines.

### 2.2. Chronic Social Defeat Stress (CSDS)

The CSDS mouse model was induced following an established protocol with minor modifications [20]. Screening: Prior to the first social defeat, retired CD-1 mice underwent a 3-day screening for aggressive behavior. They were housed individually in large plastic cages (32 cm × 21.5 cm × 17 cm) fitted with partitions, and remained in this configuration throughout the social defeat stress period. Social defeat commenced 24 h after the conclusion of the final day of screening. Chronic social defeat stress: Experimental mice were introduced into the home cage of an unfamiliar aggressive CD-1 mouse and subjected to direct physical interaction for 5–10 min. Subsequently, the test mice were physically separated from the aggressor by a perforated transparent partition; this prevented further physical contact but allowed sensory cues to continue inducing psychological stress for 24 h, until they were re-exposed to a different aggressor. The social defeat experiment lasted 10 days, with a new CD-1 aggressor mouse used each day to prevent the subjected mice from adapting to or becoming familiar with the aggressor. Control mice were housed in pairs on either side of the partition in the same manner and were never exposed to CD-1 mice. Following the final social defeat session, all mice were housed individually in new standard cages.

### 2.3. Social Interaction Test (SIT)

The social interaction test was performed as previously described [21]. Behavioral testing was carried out in a square open-field chamber measuring 50 × 50 × 50 cm. The test comprised two consecutive 2.5 min sessions. During the first session (without a social target), the experimental mouse was allowed to freely explore the arena containing an empty wire-mesh cage (12 cm × 9 cm × 12.5 cm) positioned at the center of one side of the field. During the second session (target present), the procedure was identical except that a novel CD-1 mouse, which had not previously participated in the social defeat procedure, was placed inside the wire-mesh cage. A virtual interaction zone (18 cm × 28 cm) was defined surrounding the wire-mesh cage. In both sessions, the time spent in the defined zones was recorded and analyzed using Anymaze software (version 5.21, Stoelting Co., Wood Dale, IL, USA). Social interaction performance was assessed using the social interaction ratio, calculated as the duration spent in the interaction zone with a target mouse relative to that without a target mouse.

### 2.4. Experimental Design

The probiotic strain *L. plantarum* MNN was cultured overnight for 16 h at 37 °C in MRS broth (Haibo, Qingdao, China). The pellet was recovered via centrifugation at 8000 rpm for 3 min, after which it underwent three successive washes with PBS. The MNN strain was resuspended in PBS buffer. The probiotic was activated once daily, and freshly activated strains were administered via oral gavage on each occasion. Specifically, 100 μL of the bacterial culture was inoculated into fresh MRS broth and incubated at 37 °C until culture reached the logarithmic growth phase, ensuring that actively growing bacteria were used for subsequent experiments. *L. plantarum* MNN was deposited at the China General Microbiological Culture Collection Center (CGMCC) under accession number CGMCC No. 31005.

To evaluate the effects of *L. plantarum* MNN, the prebiotic GOS, and synbiotic pretreatment on stress-induced behavioral deficits, mice were randomly divided into five experimental groups (*n* = 6): the control group (C), the stress model group (M), the stress group pretreated with MNN (MNN), the stress group supplemented with GOS (GOS) and the stress group supplemented with both MNN and GOS (MNN + GOS). Mice in the MNN and MNN + GOS groups received 200 µL of a bacterial suspension containing 1 × 10^9^ colony-forming units (CFU) daily, with PBS and a 2% GOS solution serving as vehicles, respectively; mice in the GOS group received 200 µL of a 2% (*w*/*v*) GOS solution daily (4 mg/day, equivalent to approximately 200 mg/kg/day); and mice in the control group received an equal volume of PBS solution daily. A schematic diagram of the intervention and assessment procedures is shown in Figure 1 (Figure 1A). Following acclimatization, mice received the assigned intervention once daily for 20 days before CSDS. Supplementation was initiated 20 days before CSDS exposure and continued throughout the subsequent 10-day CSDS procedure and behavioral testing, resulting in a total intervention period of 30 days. Mice underwent behavioral testing, including the Social Interaction Test (SIT), Open Field Test (OFT), Tail Suspension Test (TST) and Forced Swim Test (FST), and were euthanized 24 h after the conclusion of the behavioral assessments. Following collection via orbital puncture, blood samples were centrifuged at 5000 rpm at 4 °C.

### 2.5. Behavioral Testing

#### 2.5.1. Open Field Test (OFT)

Mice were individually placed in a novel open-field arena (50 cm × 50 cm × 50 cm) and allowed to explore freely for 6 min. A central zone (25 cm × 25 cm) was defined in the middle of the arena. After each experiment, the experimental area was disinfected with 75% alcohol and then dried with paper towels to avoid odor interference in mice. Subsequently, the mice were replaced, and the experiment is repeated [22]. The duration in the central zone, the number of central zone entries, and the total travel distance of mice were automatically recorded by a video tracking system (Anymaze 5.21, Stoelting, USA).

**Figure 1:**
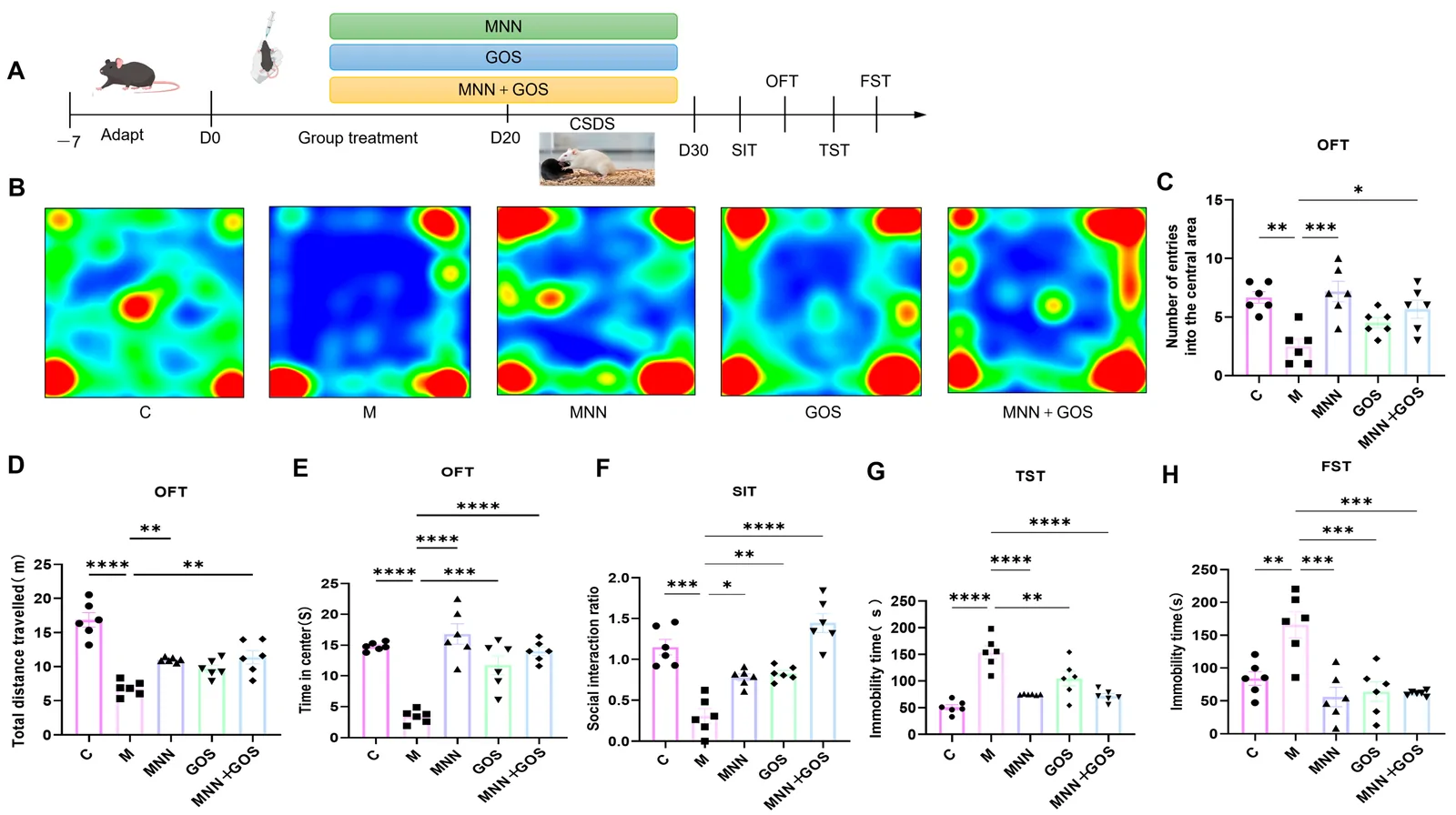
Intervention with MNN and GOS alleviates CSDS-induced abnormal stress behavioral in mice. (**A**) Schematic timeline of animal experiment. Four behavioral tests including social interaction test (SIT), open field test (OFT), tail suspension test (TST) and forced swimming test (FST) were conducted sequentially. (**B**–**E**) Indicators of open field test (**B**); representative heatmaps of locomotion trajectories in each group (**C**); number of entries into the central zone (**D**); total traveling distance (**E**); residence time in central area. (**F**) Social interaction ratio obtained from SIT. (**G**) Immobility time in TST. (**H**) Immobility time in FST. Data are expressed as mean ± SEM. Statistical analysis was performed via one-way ANOVA followed by Tukey’s post hoc test, * *p* < 0.05, ** *p* < 0.01, *** *p* < 0.001, and **** *p* < 0.0001.

#### 2.5.2. Tail-Suspension Test (TST)

The tail suspension test (TST) was adopted to assess depressive-like behaviors of mice, following the experimental protocol reported in a prior publication [23]. Briefly, adhesive tape was wrapped around the mouse tail roughly 2 cm from the tip to suspend the animals from a horizontal grid bar positioned 30 cm above the floor for 6 min. The entire testing session was recorded using a camera, and the immobility time was analyzed using a video tracking system (Anymaze 5.21, Stoelting, USA).

#### 2.5.3. Forced Swimming Test (FST)

In the forced swimming test (FST), mice were subjected to an inescapable swimming environment to assess depression-like behavior. The testing device comprised a transparent cylindrical glass tank with a height of 35 cm and an internal diameter of 30 cm. The container was filled with temperature-controlled water (24 ± 0.5 °C) to a depth of 21.5 ± 1.5 cm. Mice were individually placed in the water and allowed to swim for 6 min. Upon completion of the test, mice were carefully retrieved from the water, towel-dried, and transferred to a warm enclosure to prevent hypothermia. Increased immobility time was considered indicative of enhanced depression-like behavior. Immobility time was recorded and analyzed using a video tracking system (Anymaze 5.21, Stoelting, USA).

### 2.6. Enzyme-Linked Immunosorbent Assay (ELISA)

Serum serotonin (5-HT) levels in mice were detected by competitive enzyme-linked immunosorbent assay (ELISA) (Cat. No.: E-EL-0033, Elabscience Biotechnology Co., Ltd., Wuhan, China). Serum corticosterone (CORT) contents in mice were determined with a commercial ELISA kit (Cat. No.: MM-92891001, Jiangsu Meimian Industrial Co., Ltd., Yancheng, Jiangsu, China). All experimental operations were performed in strict accordance with the manufacturer’s instructions.

### 2.7. Immunofluorescence

Tissues from the colon and hippocampus were fixed in 4% paraformaldehyde, embedded in paraffin, and sectioned at a thickness of 4 μm. The sections were sequentially deparaffinized in xylene and rehydrated through a series of graded ethanol solutions. After heat-induced antigen retrieval in citrate buffer (pH 6.0), the sections were allowed to cool to room temperature and blocked with 5% bovine serum albumin (BSA) for 1 h to minimize non-specific antibody binding. For immunofluorescence analysis, sections were incubated overnight at 4 °C with primary antibodies against 5-HT, ZO-1, or Claudin-1. After three washes with PBS, the sections were incubated with appropriate fluorophore-conjugated secondary antibodies. Nuclei were counterstained with DAPI, and fluorescent signals were visualized using a laser scanning confocal microscope. Immunofluorescence staining for 5-HT was performed in both colonic and hippocampal tissues, whereas ZO-1 and Claudin-1 staining was conducted specifically in colonic tissues to assess intestinal epithelial barrier integrity. The fluorescence intensity of target proteins was quantified using ImageJ(1.54g) software.

### 2.8. Western Blotting

Approximately 30 mg of clean colonic tissues were collected and placed into 2 mL round-bottom centrifuge tubes preloaded with two steel beads. Then, 300 μL SDS lysis buffer and protease inhibitor cocktail (Cat. No. P1005, Beyotime Biotechnology, Shanghai, China) were added. The samples were homogenized at 50 Hz for 15 s per cycle, with a total of 5 cycles. Afterwards, the samples were lysed on ice for 30 min and centrifuged at 10,000 rpm for 15 min. The supernatant was transferred to 1.5 mL Eppendorf tubes, and the protein concentration was determined using the BCA Protein Assay Kit (Beyotime, Shanghai, China).

The tissue lysates were mixed with loading buffer (Beyotime, Shanghai, China) and boiled for protein denaturation. Subsequently, the samples were loaded onto a 10% SDS-polyacrylamide gel. Proteins were electrotransferred onto polyvinylidene difluoride (PVDF) membranes (Millipore, Burlington, MA, USA) using a semi-dry transfer system (Bio-Rad Laboratories, Hercules, CA, USA). The membranes were blocked with 3% bovine serum albumin (BSA) in TBST buffer (10 mmol/L Tris-base, pH 7.6; 150 mmol/L NaCl; 0.1% (*v*/*v*) Tween 20) at room temperature for 2 h. After blocking, the membranes were incubated with diluted primary antibodies overnight at 4 °C. Following rinsing with TBST buffer, the membranes were incubated with secondary antibodies diluted at 1:13,000 in TBST for 2 h at room temperature. Finally, protein bands were visualized using a chemiluminescent HRP substrate (Millipore, USA).

The primary antibodies used for Western blot analysis were listed as follows: β-actin (Proteintech, Wuhan, China, Cat. No. 81115-1-RR); IDO1 (Abcam Trading Co., Ltd., Shanghai, China, Cat. No. ab311847); TPH1 (Abcam Trading Co., Ltd., Shanghai, China, Cat. No. ab52954); TPH2 (Abcam Trading Co., Ltd., Shanghai, China, Cat. No. ab184505).

### 2.9. 16S rRNA Sequence Analysis

Cecal samples were collected under sterile conditions for 16S rRNA gene sequencing profiling. Approximately 50 mg of cecal contents was collected from each mouse using sterile instruments and transferred into sterile tubes. Samples were immediately placed on liquid nitrogen and transferred to the laboratory within 10 min, followed by storage at −80 °C until DNA extraction. The time interval between sample collection and freezing was less than 5 min. Microbial genomic DNA was extracted from the cecal contents using the E.Z.N.A.^®^ Soil DNA Kit (Omega Bio-tek, Norcross, GA, USA) according to the manufacturer’s instructions. DNA quality was assessed by 1% agarose gel electrophoresis, and DNA concentration and purity were determined using a NanoDrop 2000 spectrophotometer (Thermo Scientific, Waltham, MA, USA). The V3–V4 hypervariable region of the bacterial 16S rRNA gene was amplified using the primer pair 338F (5′-ACTCCTACGGGAGGCAGCAG-3′) and 806R (5′-GGACTACHVGGGTWTCTAAT-3′). PCR amplification was performed using FastPfu DNA polymerase. The PCR products were purified and quantified before library preparation and paired-end sequencing on an Illumina sequencing platform by Majorbio Bio-Pharm Technology Co., Ltd. (Shanghai, China).

Raw paired-end sequencing reads were first demultiplexed according to sample-specific barcode sequences and subjected to quality control using fastp v0.19.6. Reads were trimmed when the average quality score fell below 20 within a 50 bp sliding window, and reads shorter than 50 bp after quality trimming or containing ambiguous bases (N) were discarded. The filtered paired-end reads were merged using FLASH, requiring a minimum overlap of 10 bp and allowing a maximum mismatch ratio of 0.2 within the overlapping region. Reads that could not be successfully merged were discarded.

The resulting high-quality sequences were clustered into operational taxonomic units (OTUs) using UPARSE v7.1 at a 97% sequence similarity threshold. Chimeric sequences were removed during the OTU clustering process. Sequences assigned to chloroplasts and mitochondria were also removed from the OTU table. To minimize the potential influence of unequal sequencing depth on subsequent diversity analyses, sequences were rarefied to 20,000 reads per sample. The average Good’s coverage after rarefaction was 99.09%.

Taxonomic classification of representative OTU sequences was performed using the RDP Classifier (v2.11) against the SILVA 16S rRNA gene database (v138), with a confidence threshold of 70%. The resulting OTU table was used for downstream analyses of microbial community composition and diversity.

Alpha diversity indices, including Chao1, Simpson and Shannon indices, were calculated using Mothur. Beta diversity was assessed based on Bray–Curtis dissimilarities, followed by principal coordinate analysis (PCoA). Differences in microbial community structure among experimental groups were further evaluated using PERMANOVA. Differentially abundant taxa were identified using LEfSe with an LDA score threshold of >2.0. Based on the 16S rRNA gene sequencing data, PICRUSt2 (v2.2.0) was applied to predict the functional abundance of microbial KEGG Pathway Level 3. Differentially abundant functional pathways were identified using the Kruskal–Wallis rank-sum test. *p* values were adjusted with the Benjamini–Hochberg procedure to correct for multiple comparisons, with a false discovery rate (FDR) of 0.05. The abundance matrix of core pathways was subjected to row-wise Z-score normalization, and a heatmap was generated using GraphPad Prism 9 (La Jolla, CA, USA) to visualize the differences in functional profiles among groups.

### 2.10. LC-MS/MS Analysis

LC-MS/MS detection was carried out using a Nexera Series LC-40 liquid chromatography system coupled to a QTRAP^®^ 6500+ mass spectrometer (Sciex, Framingham, MA, USA) at Ma-jorbio Bio-Pharm Technology Co., Ltd. (Shanghai, China). Chromatographic separation was performed on an ACQUITY UPLC^®^ HSS T3 column (2.1 mm × 150 mm, 1.8 μm) maintained at 40 °C, with an 18 min gradient elution program delivered at a flow rate of 1 mL/min. Mobile phase A was water containing 0.1% formic acid, and mobile phase B was acetonitrile supplemented with 0.1% formic acid. The gradient elution conditions were as follows: 0.0–2.5 min, linear gradient from 1% to 11% B; 2.5–5.5 min, isocratic elution at 11% B; 5.5–6.5 min, 11–28% B; 6.5–7.5 min, 28% B maintained; 7.5–12.5 min, 28–50% B; 12.5–13.5 min, 50–95% B; 13.5–15.5 min, 95% B kept constant; 15.5–15.6 min, 95–1% B; 15.6–18 min, 1% B for column re-equilibration. The QTRAP^®^ 6500+ mass spectrometer was operated in both positive and negative ionization modes with an electrospray ionization (ESI) source. The source parameters were set as follows: source temperature of 550 °C, curtain gas (CUR) of 35 psi, CAD gas pressure at Medium, both Ion Source Gas1 and Gas2 at 50 psi, and ion-spray voltage floating (ISVF) of 5500 V (positive mode)/−4500 V (negative mode).

### 2.11. Statistical Analysis

All data are expressed as the mean ± Standard Error of the Mean (SEM). Statistical calculations and comparative analyses were performed with GraphPad Prism 9.0.0 software (GraphPad Software, San Diego, CA, USA). Differences among multiple groups were analyzed by one-way analysis of variance (ANOVA) followed by Tukey’s post hoc multiple comparisons test. A *p* value < 0.05 was considered statistically significant. All experiments were conducted with three independent biological replicates.

## 3. Results

### 3.1. MNN, GOS, and Synbiotic Treatments Ameliorate Stress-Induced Behavioral Abnormalities in CSDS Mice

Behavioral assessments were performed on day 31 to determine whether MNN, GOS, and the synbiotic alleviated CSDS-induced behavioral abnormalities (Figure 1). Representative heatmaps of the OFT clearly showed that mice in the model group exhibited a remarkable reduction in overall exploratory activity and a strong tendency to remain in the peripheral areas, whereas mice receiving MNN, GOS or the synbiotic showed a trend towards increased exploratory behaviour in the central area (Figure 1B). In particular, mice in the model group exhibited a notable reduction in central area entry counts, a decline in central zone residence time, and a shorter total distance travelled. Compared with the M group, MNN, GOS and the synbiotic significantly increased the number of entries into the central area, increased the total distance travelled, and prolonged the time spent in the central area (Figure 1C–E). The results of the SIT experiment showed that, compared with the control group (C), the social interaction rate of mice in the M group was significantly reduced (Figure 1F), whereas the social interaction rates of mice receiving MNN, GOS or synbiotic were all significantly increased, with the combined intervention group exhibiting the highest social interaction rate. In the TST, the immobility time of the model mice was markedly longer than that of the control group; in contrast, MNN, GOS and synbiotic pretreatment all significantly reduced immobility time (Figure 1G). Finally, in the FST, the immobility time of the model mice was markedly longer than that of the control group, whereas all three pretreatment groups significantly reduced immobility time (Figure 1H). The detailed numerical data are provided in Supplementary Table S1.

### 3.2. Effects of MNN, GOS, and Synbiotic Treatments on Gut Microbiome Composition in CSDS Mice

Given that stress induces gut microbiota dysbiosis, we assessed the effects of MNN, GOS and the synbiotic on the gut microbiota in CSDS mice. As demonstrated by the results, the Chao, Shannon and Simpson indices were markedly decreased in the M group. In comparison with the control group, three interventions reversed these alterations, with varying magnitudes of effect. Specifically, the synbiotics group exhibited the highest Chao index, only GOS increased the Shannon index, and both the MNN and synbiotics groups showed comparably elevated Simpson indices (Figure 2A–C). PCoA based on Bray–Curtis dissimilarities at the OTU level showed distinct clustering patterns between the different groups (Figure 2D). PERMANOVA further confirmed that the overall gut microbial community composition differed significantly among the groups (R^2^ = 0.3188, *p* = 0.001). Specifically, the synbiotics group exhibited the highest Chao index, only GOS increased Shannon index, and both the MNN and synbiotics groups showed comparably elevated Simpson indices. In particular, The M and C groups were clearly separated along both the PC1 and PC2 axes. Following MNN, GOS and synbiotic interventions, the distribution of the microbiota shifted markedly away from the M group and moved closer to the C group.

Notably, at the genus level, further screening identified representative bacterial genera exhibiting differential abundance, all the aforementioned genera exhibited LDA scores above 2.0 (Figure 2F–L). The results showed that *Ligilactobacillus* had the highest LDA score and was the characteristic genus of Group C. Relative to the control group, the microbial composition of the model group (M) exhibited a marked shift, characterised primarily by the significant enrichment of *Mucispirillum* and *Oscillibacter*. The GOS group exhibited a unique community composition, characterised by the high-abundance enrichment of *Odoribacter*. The microbial profile of the MNN group was primarily distinguished by the significant enrichment of *Rikenella*. Under the combined effects of MNN and GOS, the gut microbiota composition underwent further remodelling, with *Lachnospiraceae_UCG-006* and *[Eubacterium]_siraeum_group* becoming significantly dominant.

The functional potential of the gut microbiome was inferred based on 16S rRNA gene sequencing data via the PICRUSt2 bioinformatic tool. Twenty core differentially abundant KEGG Pathway Level 3 pathways (Kruskal–Wallis test, raw *p* < 0.05) were visualized using a heatmap with row-wise Z-score normalization(Figure 3). Results showed that the predicted functional abundances of tryptophan metabolism and serotonergic synapse pathways in gut microbiota were relatively lower in the model group. MNN supplementation markedly upregulated the functional abundance of both pathways, suggesting that MNN may modulate the tryptophan metabolic potential of gut microbiota and thereby participate in the regulation of 5-HT-related neural signaling, while the GOS and MNN + GOS groups selectively enriched potential functions related to nutrient metabolism and cellular signal transduction.

**Figure 3:**
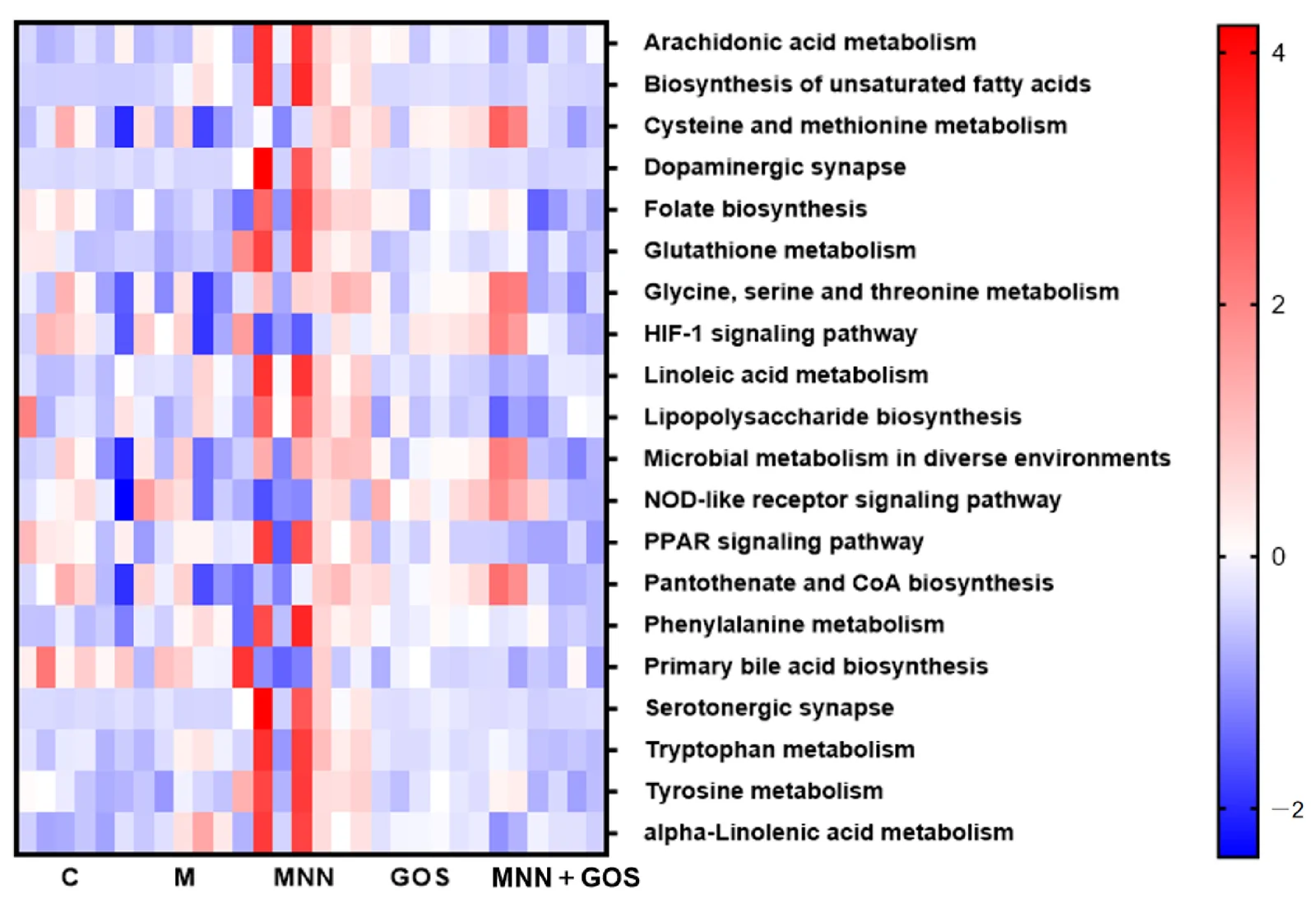
Heatmap of differentially abundant KEGG Pathway Level 3 pathways predicted by PICRUSt2. The abundance of each pathway was subjected to row-wise Z-score normalization. The color gradient represents the Z-score value, where blue indicates lower pathway abundance and red indicates higher abundance relative to the overall mean across all samples. Each group contained 6 biological replicates. Full names of KEGG pathways are listed on the right side of the heatmap. Differential abundance was determined by Kruskal–Wallis rank-sum test. Differential abundance was determined by the Kruskal–Wallis rank-sum test, followed by Benjamini–Hochberg correction for multiple comparisons, with an FDR < 0.05 set as the significance threshold.

### 3.3. MNN, GOS and MNN + GOS Remodel Tryptophan Metabolism in CSDS Mice

To further elucidate the effects of CSDS and the three interventions on in vivo tryptophan metabolism, this study utilised mouse caeca for targeted metabolomics analysis. Relative to the control group, the analytical results indicated that CSDS significantly altered the balance of tryptophan metabolism. Specifically, metabolites associated with the kynurenine pathway (Kyn, 3-hydroxykynurenine [3-HK] and quinolinic acid [QA]) were significantly elevated in the CSDS group; MNN, GOS and MNN + GOS restored the levels of these metabolites to varying degrees; in contrast, levels of metabolites in the 5-HT metabolic pathway—such as 5-HTP, 5-HT and 5-hydroxyindole-3-acetic acid (5-HIAA)—were significantly reduced in the CSDS group. All three interventions restored these metabolites to varying degrees. In particular, the combined MNN + GOS preventive intervention produced the highest 5-HTP/Trp ratio and markedly increased 5-HIAA, while GOS produced the greatest increase in cecal 5-HT (Figure 4G,L,N). These results indicate that MNN and GOS are capable of rebalancing the kynurenine and 5-HT metabolic pathways within tryptophan metabolism. The Kyn/Trp ratio was markedly elevated by CSDS and was partially reduced by MNN and GOS. In contrast, the ratio remained elevated in the MNN + GOS group despite the concomitant increase in the 5-HTP/Trp ratio (Figure 4M,N). Thus, the combined intervention did not simply shift tryptophan metabolism away from the kynurenine path-way; rather, it appeared to enhance tryptophan utilization through the serotonin branch while altering the downstream distribution of kynurenine metabolites. Among the microbiota-derived indole metabolites, CSDS markedly increased IS, which was restored by MNN, GOS and MNN + GOS. Meanwhile, CSDS decreased ILA, which was restored by MNN and GOS but not MNN + GOS. IPA did not differ significantly among the groups (Figure 4P), whereas MNN increased IAA relative to the model group (Figure 4Q).

To further clarify the regulatory interaction between the gut microbiome and tryptophan metabolism, alongside its association with stress-related behavioral phenotypes, we performed Spearman’s correlation analysis to systematically evaluate the relationships among differentially abundant bacterial genera, key tryptophan metabolites, and behavioral indicators(Figure 5). In the 5-HT pathway, the stress-related genus *Mucispirillum* was significantly negatively correlated with 5-HT (r = −0.700, *p* < 0.01), while *Odoribacter* showed a significant positive correlation with 5-HT (r = 0.525, *p* < 0.05). *Ligilactobacillus* was positively correlated with 5-HT precursor 5-HTP, and *Rikenella* was positively correlated with 5-HT degradation product 5-HIAA. In the KYN pathway, *Oscillibacter* was positively correlated with KYN levels, while *Rikenella* was strongly negatively correlated with KYN (r = −0.887, *p* < 0.001). *Mucispirillum* and *Oscillibacter* were positively associated with neurotoxic metabolites QA and 3-HK, whereas neuroprotective metabolite KYNA was negatively correlated with *Oscillibacter* and positively correlated with *Rikenella*. For behavioral indicators, *Ligilactobacillus* was positively associated with SIT ratio and negatively correlated with TST immobility time. *Oscillibacter* and *Rikenella* showed opposite correlations with OFT.

**Figure 4:**
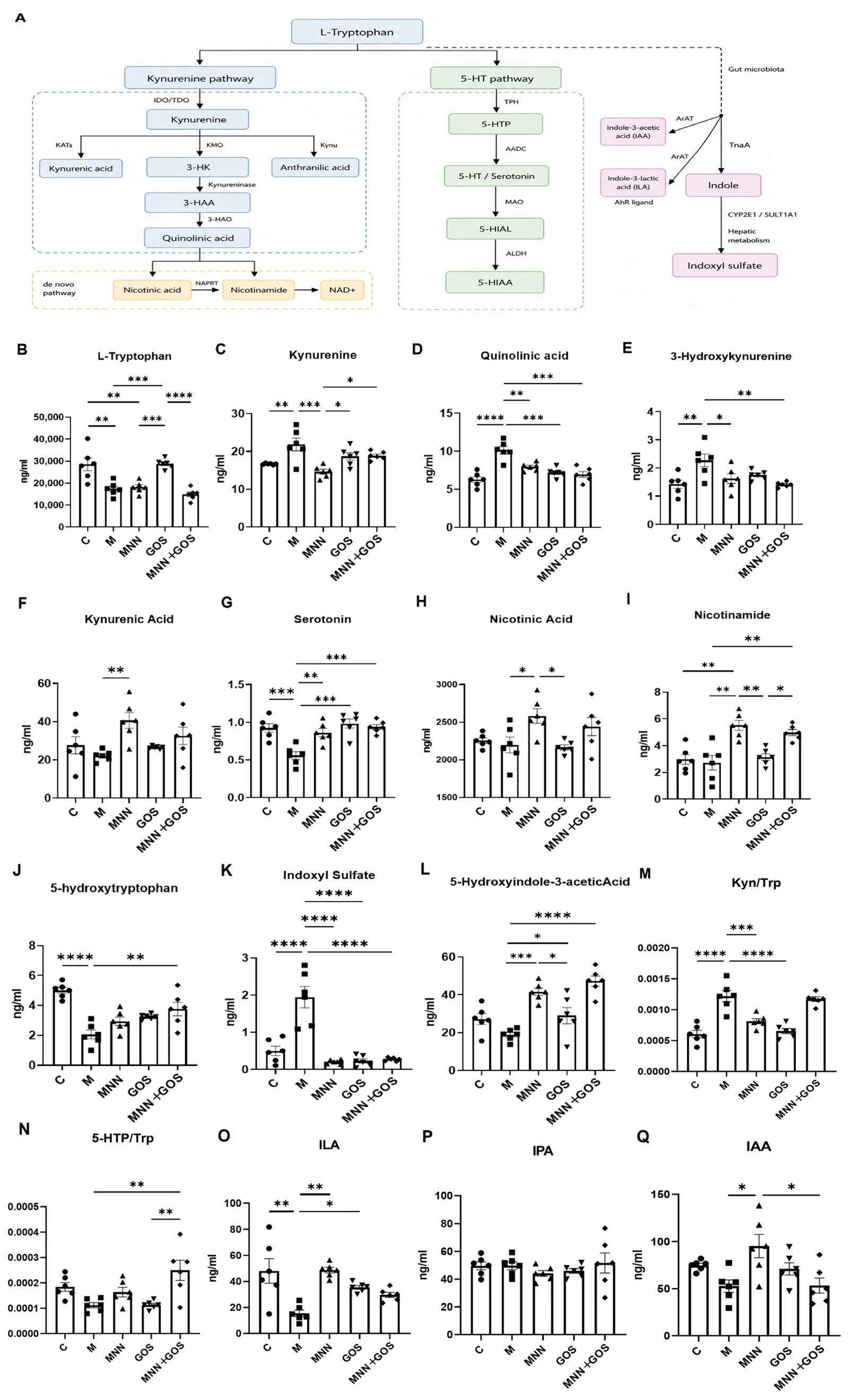
Tryptophan metabolism and its regulation in the cecum. (**A**) Schematic representation of tryptophan metabolic pathways in the cecum, highlighting the bifurcation toward the kynurenine, serotonin. (**B**–**Q**) Relative concentrations of key tryptophan metabolites in cecal content (**B**), L-tryptophan (**C**), kynurenine (**D**), quinolinic acid (**E**), 3-hydroxykynurenine (**F**), kynurenic acid (**G**), serotonin (**H**), nicotinic acid (**I**), nicotinamide (**J**), 5-hydroxytryptophan (**K**), indoxyl sulfate (**L**), 5-hydroxyindol-3-eacetic acid (**M**), Kynurenine/Tryptophan (**N**), 5-HTP/Tryptophan (**O**), Indole-3-lactic acid (**P**), Indole-3-propionic acid (**Q**), Indole-3-acetic acid. Data are presented as mean ± SEM (n = 6). Significant differences between groups are indicated by asterisks: * *p* < 0.05, ** *p* < 0.01, *** *p* < 0.001, and **** *p* < 0.0001.

**Figure 5:**
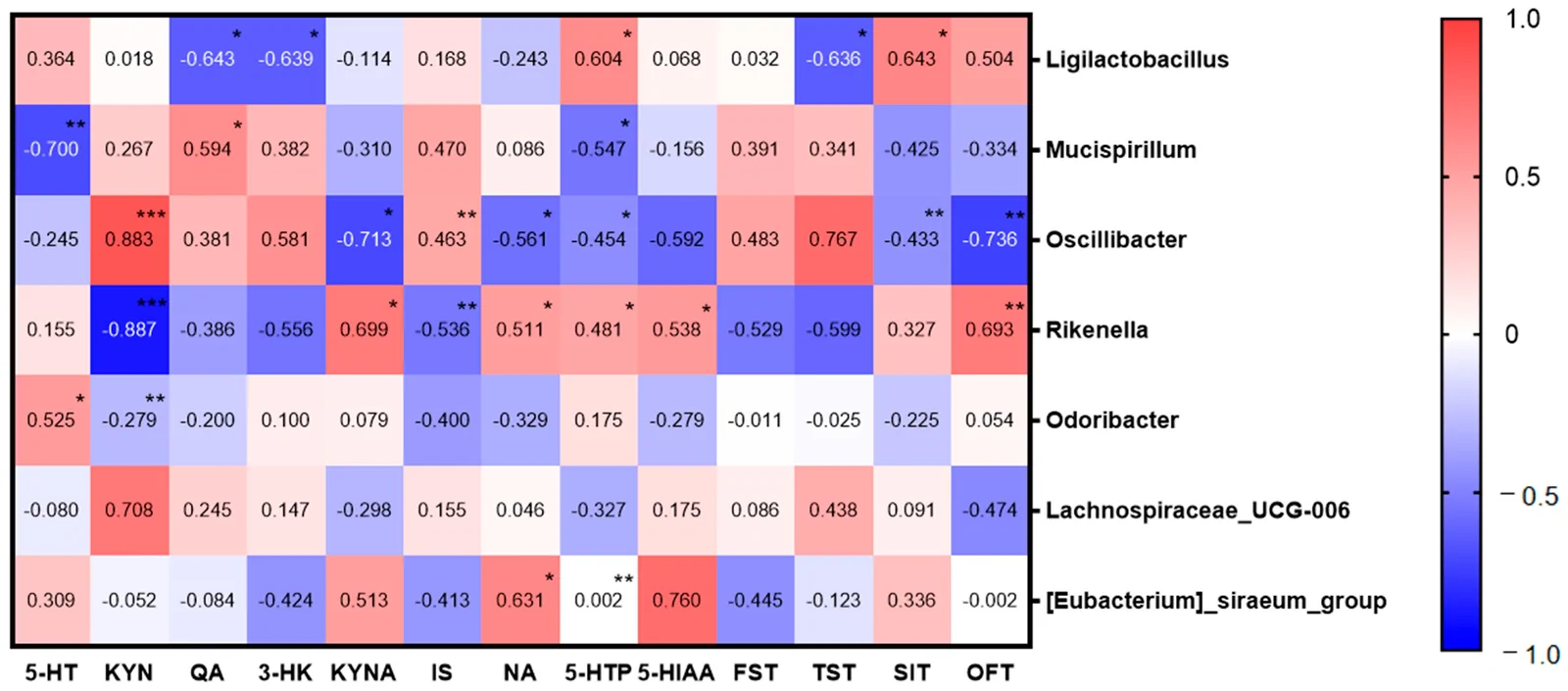
Heatmap of Spearman correlations between differentially abundant gut microbial genera, key tryptophan metabolites and behavioral indicators. Red and blue gradients indicate positive and negative correlations, respectively, with color depth representing the magnitude of the correlation coefficient. The exact correlation value is labeled in each grid. Asterisks denote statistical significance: * *p* < 0.05, ** *p* < 0.01, *** *p* < 0.001.

### 3.4. MNN, GOS, and MNN + GOS Treatments Regulate the Intestinal Tryptophan Metabolic Pathway in CSDS Mice

In this study, we conducted a comprehensive evaluation of the expression levels of IDO1 and TPH1, key enzymes involved in the kynurenine pathway and serotonin synthesis, respectively, within colonic tissue. Western blot analyses revealed a significant upregulation of IDO1 protein expression in group M compared to group C (Figure 6D), whereas TPH1 expression was notably downregulated (Figure 6E). Interventions with either MNN or GOS individually, as well as the combined MNN + GOS supplementation, effectively mitigated the aberrant overexpression of IDO1 protein (Figure 6D). Concurrently, both GOS supplementation alone and combined MNN + GOS supplementation significantly enhanced TPH1 protein expression levels (Figure 6E). Immunofluorescence and quantitative analysis of colonic 5-HT demonstrated a substantial reduction in the fluorescence intensity of 5-HT in group M compared to group C (Figure 6A,B). Subsequent supplementation with MNN, GOS, or their combination resulted in a significant restoration of 5-HT fluorescence intensity, with the most pronounced recovery observed in the MNN + GOS group, suggesting a superior protective effect of the combined intervention. Serum 5-HT levels, quantified via ELISA, were notably reduced in the model group in comparison with the control group. However, supplementation with MNN and the combined regimen led to a significant elevation in serum 5-HT levels relative to group M (Figure 6F). Furthermore, corticosterone (CORT) levels in the blood were assessed, revealing significantly elevated levels in the model group compared to the control group. Supplementation with MNN, GOS, or MNN + GOS resulted in a reduction in CORT levels, with the most substantial effects observed in the MNN and MNN + GOS groups (Figure 6G). Since intestinal barrier integrity is a critical component of the microbiota–gut–brain axis, we further examined the integrity of the colonic epithelial barrier by assessing the expression of tight junction proteins. Immunofluorescence staining revealed that the fluorescence intensities of ZO-1 and Claudin-1 were markedly reduced in the colonic tissues of group M compared with group C (Figure 6H–J), indicating impaired intestinal barrier integrity following CSDS exposure. Quantitative analysis further confirmed significant decreases in both ZO-1 and Claudin-1 expression in the model group. Conversely, administration of MNN, GOS, or MNN + GOS significantly restored the expression levels of these tight junction proteins, with the MNN + GOS group exhibiting the most pronounced improvement, suggesting that the combined intervention effectively alleviated CSDS-induced intestinal barrier disruption. The detailed numerical data are provided in Supplementary Table S1.

### 3.5. MNN, GOS, and MNN + GOS Alleviate Hippocampal 5-HT System Dysfunction and Enhance Neuroplasticity in CSDS Mice

To explore the potential mechanisms underlying 5-HT regulation, we evaluated the expression of IDO1 and TPH2, key regulatory enzymes in the tryptophan–5-HT metabolic pathway, as well as proteins related to synaptic plasticity within the hippocampus of mice (Figure 7A–G). Western blot analyses indicated a significant upregulation of IDO1 expression in the model (M) group compared to the control (C) group, while TPH2 expression exhibited a declining trend (Figure 7C–E). Immunofluorescence assays demonstrated a marked reduction in 5-HT fluorescence intensity in the hippocampus of mice subjected to the CSDS condition. Notably, supplementation with MNN, GOS, and the combined MNN + GOS effectively ameliorated these alterations. Additionally, we assessed the expression levels of BDNF and PSD95, which are indicative of neuroplasticity (Figure 7C,F,G). In comparison to Group C, the expression of BDNF and PSD95 was significantly decreased in the hippocampus of mice in Group M. Supplementation with MNN, GOS, and the combined MNN + GOS resulted in a significant restoration of BDNF and PSD95 expression, with the MNN + GOS group showing the greatest increase in PSD95 expression among the intervention groups.

## 4. Discussion

CSDS is a well-recognized psychosocial stress model widely used to recapitulate a range of stress-evoked behavioral abnormalities including social avoidance and defensive behaviors triggered by prolonged environmental stress [24]. This model has been rigorously validated in rodent studies and is a fundamental tool for elucidating the mechanisms underlying chronic stress responses and psychological stress-induced impairments [25]. From a behavioral perspective, the behavioral phenotypes induced by the CSDS model in mice share a high degree of consistency with the behavioral manifestations of chronic stress in cats. Specifically, CSDS-induced reduction in the SIT is analogous to social withdrawal [26], which strongly recapitulates the persistent hiding behavior and social aversion commonly observed in cats under chronic stress [27]. OFT is a classical behavioral assay used to assess stress levels in cats, and it is widely applied to quantify stress responses across diverse animal species following exposure to novel environments. In this study, we demonstrated that feline milk-derived *L. plantarum* MNN, GOS, and MNN + GOS improve CSDS-induced behavioral abnormalities by altering the gut microbiota and modulating tryptophan metabolism. Targeted tryptophan metabolomics combined with fecal 16S rRNA analysis revealed alterations in gut microbiota and tryptophan metabolism that were accompanied by changes in central 5-HT levels and brain tryptophan metabolism, supporting the potential involvement of the gut microbiota–tryptophan metabolism–serotonin axis in the beneficial effects of the interventions.

Under physiological conditions, tryptophan is primarily metabolized through the 5-HT and KYN pathways, which are regulated by the rate-limiting enzymes TPH1/TPH2 and IDO1, respectively [28]. Our results demonstrated that CSDS markedly increased intestinal IDO1 expression and decreased TPH1 expression, accompanied by reduced 5-HT levels, together with elevated KYN levels in mice. Both MNN and GOS significantly downregulated intestinal IDO1 and upregulated TPH1, which drives peripheral 5-HT synthesis and reduces the Kyn/Trp ratio. Interestingly, although the synbiotic group exhibited a moderately elevated Kyn/Trp ratio, the 5-HTP/Trp ratio was simultaneously increased, indicating an overall enhancement of tryptophan metabolic turnover rather than excessive activation of the KYN pathway. These findings suggest that combining MNN with GOS may more effectively restore the balance of tryptophan metabolism under chronic stress. Preservation of intestinal tryptophan may increase substrate availability for transport across the blood–brain barrier (BBB), thereby facilitating central serotonin synthesis. Consistent with this possibility, our behavioral results showed that MNN, GOS and the synbiotic alleviated stress-related behavioral abnormalities, which may be associated with improved serotonergic signaling.

Besides serotonin deficiency, excessive activation of the KYN pathway represents another important mechanism linking stress exposure to behavioral abnormalities. KYN can enter the circulation and readily cross the blood–brain barrier, where it is further metabolized into neuroactive derivatives, including QA and 3-HK [29]. These metabolites have been widely implicated in depression-like behaviors [30]. Specifically, 3-HK induces neuronal oxidative damage and synaptic dysfunction, while QA acts as an N-methyl-D-aspartate (NMDA) receptor agonist to trigger excitotoxicity and neuroinflammation [31]. Consistent with these observations, MNN, GOS, and the synbiotic intervention all reduced the levels of QA and 3-HK, suggesting that these interventions may alleviate stress-induced behavioral abnormalities by suppressing the production of neurotoxic kynurenine metabolites.

Further metabolomic analysis revealed that MNN and the synbiotic intervention promoted the conversion of downstream KYN metabolites toward nicotinamide. As nicotinamide serves as a major precursor for NAD^+^ biosynthesis [32], this metabolic shift may support the cellular energy metabolism required for efficient serotonin syn-thesis [33]. However, the contribution of this pathway to enterochromaffin (EC) cell function requires further experimental validation. In addition, gut microbiota metabolize tryptophan into a variety of indole derivatives [34]. We found that all three interventions effectively reduced the accumulation of indoxyl sulfate, a gut-derived uremic toxin associated with intestinal barrier dysfunction and neurological disorders [35]. This finding suggests that regulation of microbial tryptophan metabolism by MNN and GOS ex-tends beyond serotonin biosynthesis and may also reduce the production of potentially harmful microbial metabolites. Prior evidence shows that elevated corticosterone accelerates peripheral 5-HT turnover [36]. Consistent with this, CSDS mice in this study displayed markedly increased serum corticosterone alongside reduced circulating 5-HT concentrations. Notably, only MNN significantly restored serum 5-HT levels, which was associated with a greater attenuation of corticosterone elevation, suggesting that modulation of the stress hormone response may contribute to the restoration of serotonergic signaling.

Approximately 90–95% of peripheral 5-HT is synthesized by EC cells, and the gut microbiota is recognized as a key regulator of host serotonin production [37]. Microbial metabolites, including SCFAs and secondary bile acids, activate EC cells, enhance TPH1 expression, and promote intestinal 5-HT synthesis [38]. Accumulating evidence indicates that chronic stress disrupts gut microbial homeostasis, leading to alterations in microbial composition and function [39]; consistently, our study demonstrated that CSDS reduced gut microbial diversity and significantly reshaped the microbial community structure. It is worth noting that *Mucispirillum* [4] and *Oscillibacter* were markedly enriched in the CSDS group, consistent with previous studies [40]. Correlation analysis revealed that *Mucispirillum* and *Oscillibacter* were closely associated with key tryptophan metabolites and behavioral indices. Specifically, *Mucispirillum* was negatively correlated with 5-HT and positively correlated with QA and IS, while *Oscillibacter* showed positive correlations with KYN-pathway metabolites and negative correlations with 5-HT levels and behavioral indicators. These results suggest that alterations in *Mucispirillum* and *Oscillibacter* are associated with changes in tryptophan–KYN metabolism, which may contribute to CSDS-induced behavioral abnormalities. Additionally, *Mucispirillum* is frequently associated with epithelial dysfunction, mucosal immune activation and stress-related dysbiosis [41]. In contrast, MNN, GOS, and MNN + GOS supplementation significantly reduced the abundance of *Mucispirillum* and *Oscillibacter*. Among the three interventions, GOS exhibited the strongest inhibitory effect on *Mucispirillum*, whereas MNN most effectively suppressed *Oscillibacter*.

Interestingly, MNN preferentially enriched *Rikenella*. Previous studies have shown that tryptophan supplementation restores the abundance of *Rikenella* under chronic restraint stress, thereby ameliorating behavioral deficits [42]. GOS intervention tended to enrich *Odoribacter*, an SCFA-producing genus [43]. As SCFAs are recognized regulators of intestinal serotonin biosynthesis and host tryptophan metabolism, the enrichment of Odoribacter may partly contribute to the beneficial effects of GOS on tryptophan metabolic remodeling [44]. The MNN + GOS intervention exhibited an enrichment of *Lachnospiraceae* and *Eubacterium*. Reduced *Lachnospiraceae* abundance is associated with emotional disorders [45]. MNN + GOS significantly increased *Eubacterium*. Previous studies have shown that *Eubacterium* is associated with microbial tryptophan metabolism and may contribute to the production of indole derivatives, such as IAA, which act as AhR ligands involved in maintaining microbiota–gut–brain axis homeostasis [46]. Therefore, the enrichment of *Eubacterium* by MNN + GOS may help restore microbial tryptophan metabolism and alleviate chronic stress-induced behavioral abnormalities. Correlation analysis further revealed that *Rikenella*, *Odoribacter*, *Lachnospiraceae*, and *Eubacterium* were closely associated with tryptophan metabolites and behavioral indices, suggesting that MNN, GOS, and synbiotic intervention may improve stress-related behavioral deficits by selectively reshaping key bacterial taxa and restoring tryptophan metabolic homeostasis.

It is worth noting that peripheral 5-HT cannot cross the blood–brain barrier [44], consequently, an increase in gut 5-HT does not imply that it directly enters the brain to exert effects. However, tryptophan is the sole precursor for 5-HT synthesis, it can penetrate the BBB and reach the central nervous system (CNS), where it is converted into endogenous 5-HT under the catalysis of TPH2 [47]. Consequently, the regulation of tryptophan metabolism by the gut microbiota not only influences local 5-HT synthesis in the gut but also determines the supply of substrates entering the brain for 5-HT production [34]. Central 5-HT signaling is critically involved in the regulation of neuronal plasticity and emotional processing, partly through modulation of neurotrophic factors and synaptic proteins. BDNF and PSD95 are key regulators of neuroplasticity, and their reduced expression has been closely associated with stress-induced mood disorders [48]. In line with previous findings, CSDS in this study induced increased IDO1 expression and decreased TPH2 expression in the hippocampus, accompanied by a reduction in 5-HT fluorescence signal, suggesting that central tryptophan metabolism had also been altered [49]. To varying degrees, MNN, GOS, and MNN + GOS all downregulated IDO1 and upregulated TPH2 in the hippocampus, while restoring the levels of 5-HT, BDNF, and PSD95. In conjunction with the gut findings, this protective effect may be related to the restoration of tryptophan metabolic homeostasis in the gut–brain axis and the promotion of neurotrophic support.

Although this study systematically evaluated the effects of MNN, GOS, and their combined intervention on improving CSDS-induced behavioral abnormalities, gut microbiota dysbiosis, tryptophan metabolism disorders, and gut–brain axis dysfunction, there are still some limitations. All validations were conducted in the mouse CSDS model, and interspecies differences in gut microbiota composition and tryptophan metabolic pathways between mice and cats mean the intervention efficacy needs further verification via in vivo clinical trials in cats [50]. Furthermore, only male C57BL/6 mice were used in the CSDS model. Therefore, the effects of MNN, GOS, and synbiotic on stress-related behaviors and the gut–brain tryptophan metabolic pathway may not be generalizable to females. Future studies including both sexes are warranted.

## 5. Conclusions

Taken together, this study demonstrates that feline milk-derived *L. plantarum* MNN and its synbiotic combination with GOS effectively attenuate CSDS-induced behavioral abnormalities and are associated with restoration of gut–brain axis function and alterations in gut microbiota structure and tryptophan metabolism. This work advances our understanding of the potential association between gut microbiota alterations and tryptophan metabolism and provides preliminary evidence for the potential use of feline milk-derived probiotics and synbiotics as dietary strategies to alleviate stress-associated behavioral abnormalities, warranting further investigation in companion animals.

## Figures and Tables

**Figure 2:**
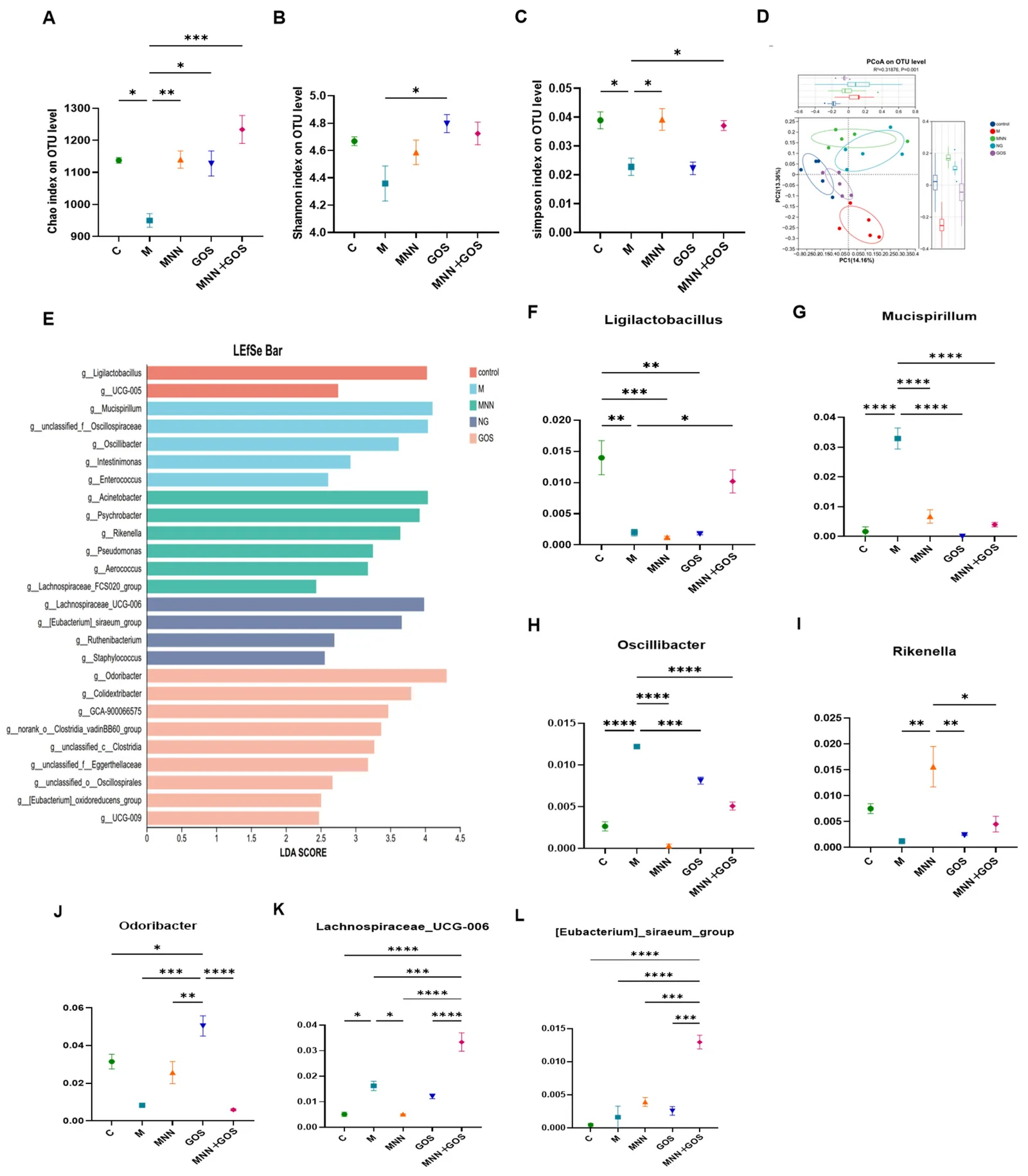
Effects of MNN and GOS on gut microbiota composition and diversity. (**A**–**C**) Alpha diversity indices at the OTU level, including (**A**) Chao1 index, (**B**) Shannon index, and (**C**) Simpson index. (**D**) Principal coordinate analysis (PCoA) plot based on the Bray–Curtis distance matrix at the OTU level, the significance of differences in overall microbial community composition among groups was assessed using PERMANOVA. (**E**) Linear discriminant analysis effect size (LEFSe) bar chart showing the significantly enriched taxonomic clades with an LDA score > 2.0. (**F**–**L**) Relative abundance of key differential bacterial genera across different groups including (**F**) Ligilactobacillus, (**G**) Mucispirillum, (**H**) Oscillibacter, (**I**) Rikenella, (**J**) Odoribacter, (**K**) Lachnospiraceae_UCG-006, (**L**) [Eubacterium]_siraeum_group. Data are presented as mean ± SEM. Significant differences between groups are indicated by asterisks: * *p* < 0.05, ** *p* < 0.01, *** *p* < 0.001, and **** *p* < 0.0001.

**Figure 6:**
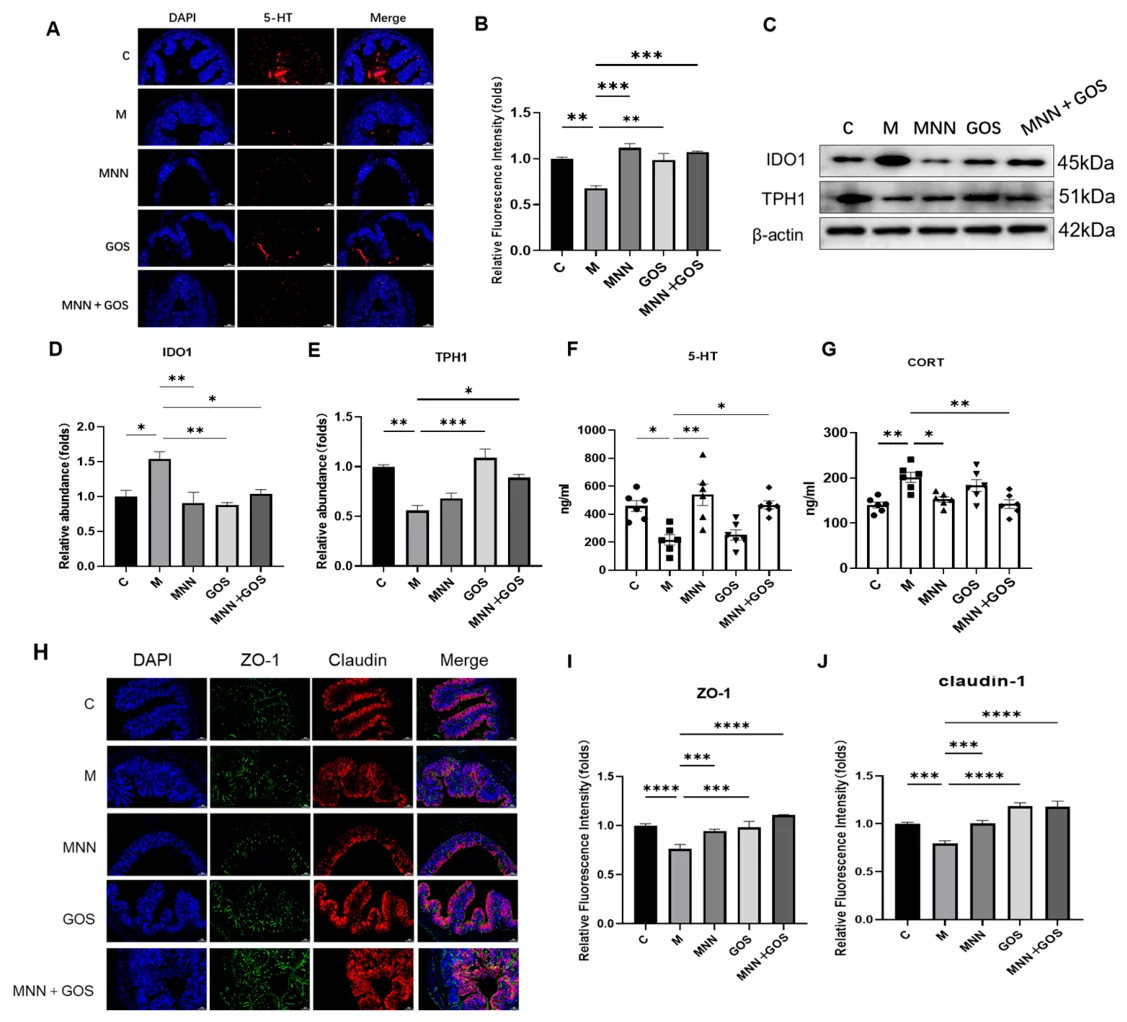
Effects of MNN, GOS, and MNN + GOS supplementation on colonic tryptophan–serotonin metabolism, stress response, and intestinal barrier integrity in CSDS mice. (**A**) Representative immunofluorescence images of 5-HT (red) and DAPI (blue) counterstaining in the colon tissue (scale bar = 100 um). (**B**) Quantification of the relative fluorescence intensity of 5-HT. (**C**) Representative Western blot bands of IDO1, TPH1, and β-actin in the colon tissue. Relative protein expression levels quantified for (**D**) IDO1 and (**E**) TPH1 normalized to β-actin. (**F**) Serum 5-HT concentration detected by ELISA. (**G**) Serum CORT concentrations across groups. (**H**) Representative immunofluorescence images showing the expression and localization of ZO-1 and Claudin-1 in colonic tissues. Blue fluorescence represents DAPI-stained nuclei, whereas green and red fluorescence indicate ZO-1 and Claudin-1 signals, respectively (scale bar = 100 μm). (**I**,**J**) Quantitative analysis of ZO-1 and Claudin-1 fluorescence intensity. Data are presented as mean ± SEM. * *p* < 0.05, ** *p* < 0.01, *** *p* < 0.001, and **** *p* < 0.0001.

**Figure 7:**
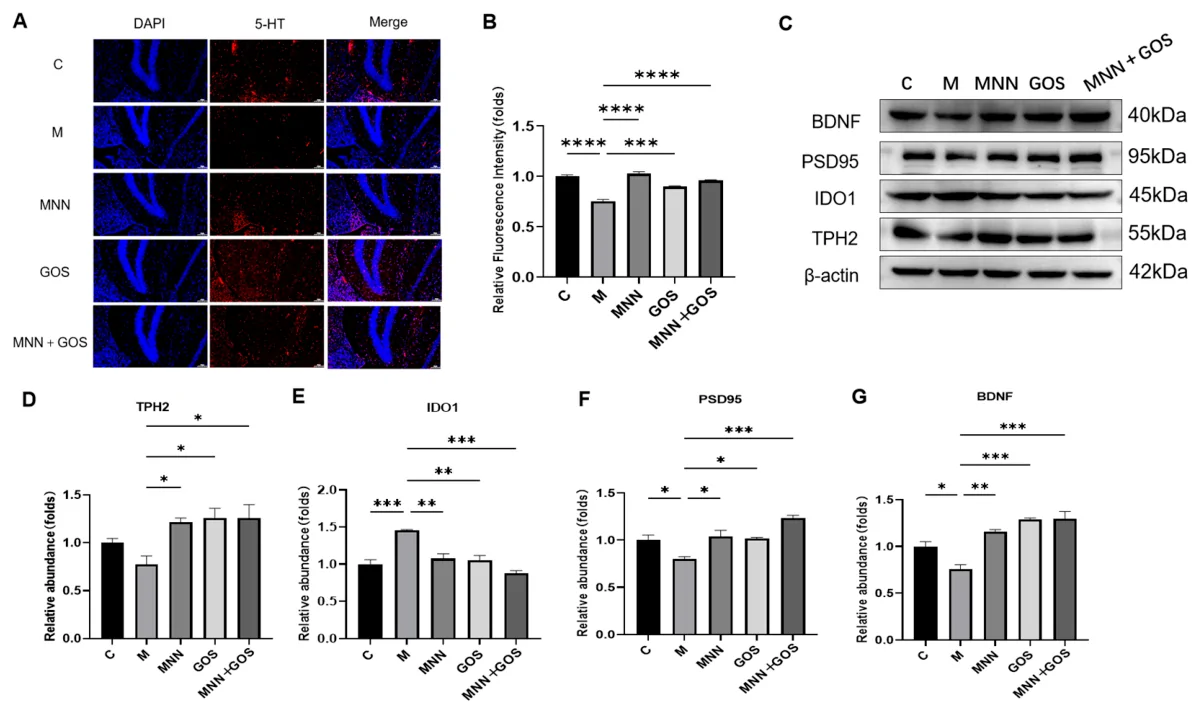
Modulation of the Trp-5-HT pathway and neurotrophic factors in the hippocampal tissue across different groups. (**A**) Representative immunofluorescence images of 5-HT positive cells (red) and DAPI (blue) counterstaining in the hippocampal tissue (scale bar = 100 um). (**B**) Quantification of the relative fluorescence intensity of 5-HT. (**C**) Representative Western blot bands showing the expression levels of BDNF, PSD95, IDO1, TPH2, and β-actin in the hippocampal tissue. The relative protein expression levels of (**D**), TPH2 (**E**), IDO1 (**F**), PSD95 and (**G**), BDNF were quantified and normalized to β-actin. Data are presented as mean ± SEM. * *p* < 0.05, ** *p* < 0.01, *** *p* < 0.001, and **** *p* < 0.0001.

## Data Availability

The original contributions presented in this study are included in the article. Further inquiries can be directed to the corresponding author.

## Supplementary Materials

The following supporting information can be downloaded at: https://www.mdpi.com/article/10.3390/microorganisms14102185/s1, Table S1: Behavioral, serum 5-HT and corticosterone data.
