# Supplementary material for: Feline Milk-Derived Lactiplantibacillus plantarum MNN and Galacto-Oligosaccharides Ameliorate Stress-Related Behavioral Abnormalities and Alter Tryptophan Metabolism in CSDS Mice
Source: Microorganisms. 2026 Sep 30;14(10):2185. doi: 10.3390/microorganisms14102185 (PMC13707920; doi:10.3390/microorganisms14102185)
Supplement: Supplementary file 1 [file microorganisms-14-02185-s001.zip › microorganisms-4517168-supplementary.pdf]

Supplementary:

Table S1: Behavioral, serum 5-HT and corticosterone data.

**Paramater**	**Group**	**Mean**	**SEM**	**ANOVA F(df)**	**P-value**
Total distance travelled in OFT（m）	C	16.85	1.063	F(4, 25) = 25.87	p < 0.0001
	M	6.811	0.4385		
	MNN	11	0.135		
	GOS	9.671	0.535		
	MNN + GOS	11.39	0.9814		
Time in center in OFT（S）	C	14.78	0.2725	F(4, 25) =23.30	p < 0.0001
	M	3.417	0.4301		
	MNN	16.80	1.649		
	GOS	11.73	1.536		
	MNN + GOS	14.00	0.6928		
5-HT (ng/ml)	C	457.9	39.5	F(4, 25) =9.198	P=0.0001
	M	216.0	37.53		
	MNN	538.4	77.44		
	GOS	251.1	36.26		
	MNN + GOS	465.9	29.1		
CORT (ng/ml)	C	140.4	6.629	F(4, 25) =7.769	P=0.0003
	M	201.7	11.29		
	MNN	152.6	6.004		
	GOS	183.6	13.27		
	MNN + GOS	142.3	9.643		
